# Diagnostic delay in symptomatic uncomplicated diverticular disease: an Italian tertiary referral centre study

**DOI:** 10.1007/s11739-023-03446-x

**Published:** 2023-10-27

**Authors:** Giovanni Santacroce, Marco Vincenzo Lenti, Giulia Maria Abruzzese, Giacomo Alunno, Francesco Di Terlizzi, Carmine Frenna, Antonella Gentile, Mario Andrea Latorre, Clarissa Petrucci, Damiano Ruggeri, Simone Soriano, Nicola Aronico, Carlo Maria Rossi, Annalisa De Silvestri, Gino Roberto Corazza, Antonio Di Sabatino

**Affiliations:** 1 Department of Internal Medicine and Medical Therapeutics, University of Pavia, Pavia, Italy; 2 First Department of Internal Medicine, San Matteo Hospital Foundation, Pavia, Italy; 3 Clinical Epidemiology and Biometric Unit, San Matteo Hospital Foundation, Pavia, Italy

**Keywords:** Diverticular disease, Irritable bowel syndrome, Misdiagnosis, Patient education

## Abstract

The magnitude of the diagnostic delay of symptomatic uncomplicated diverticular disease (SUDD) is unknown; we aimed to evaluate SUDD diagnostic delay and its risk factors. SUDD patients diagnosed at a tertiary referral centre were retrospectively enrolled (2010–2022). Demographic and clinical data were retrieved. Overall, patient-, and physician-dependant diagnostic delays were assessed. Univariate and multivariate analyses were fitted to identify risk factors for diagnostic delay. Overall, 70 SUDD patients (median age 65 years, IQR 52–74; F:M ratio = 1.6:1) were assessed. The median overall diagnostic delay was 7 months (IQR 2–24), patient-dependant delay was 3 months (IQR 0–15), and physician-dependant delay was 1 month (IQR 0–6). Further, 25% of patients were misdiagnosed with irritable bowel syndrome (IBS). At multivariate analysis, previous misdiagnosis was a significant risk factor for overall and physician-dependant diagnostic delay (OR 9.99, p = 0.01, and OR 6.46, p = 0.02, respectively). Also, a high educational level (> 13 years) was associated with a greater overall diagnostic delay (OR 8.74 p = 0.02), while previous abdominal surgery was significantly associated to reduced physician-dependant diagnostic delay (OR 0.19 p = 0.04). To conclude, SUDD may be diagnosed late, IBS being the most frequent misdiagnosis. Timely diagnosis is crucial to tackle the burden of SUDD on patients and healthcare.

**Supplementary Information:**

The online version contains supplementary material available at 10.1007/s11739-023-03446-x.

## Introduction

Approximately 25% of individuals with diverticulosis may experience symptoms, including bloating, abdominal pain, and changes in bowel habits, without endoscopic signs of inflammation. These features define a condition known as symptomatic uncomplicated diverticular disease (SUDD) [1]. SUDD significantly impacts patients’ quality of life, due to its chronic behaviour and disabling symptoms [2, 3]. Furthermore, the risk of progression to diverticulitis, leading to complications and disease recurrence [4], constitutes a burden on the healthcare systems. A prompt diagnosis is therefore crucial for effective disease management. However, the non-specific symptoms associated with SUDD, which often overlaps with other conditions like irritable bowel syndrome (IBS) [5], may result in a challenging diagnosis, especially in young adults, leading to diagnostic delay.

Although diagnostic delay has been evaluated in other chronic disorders of the gastrointestinal tract, such as coeliac disease, autoimmune gastritis, eosinophilic esophagitis, and inflammatory bowel disease (IBD) [6–9], there is a notable lack of data regarding diagnostic delay in SUDD. Therefore, the primary objective of our study was to assess the overall, patient-dependant and physician-dependant diagnostic delay in patients with SUDD. Additionally, we sought to investigate potential factors contributing to prolonged diagnostic delay.

## Materials and methods

### Study population and design

This retrospective and single-centre study was conducted at a tertiary referral centre of Northern Italy (San Matteo Hospital Foundation), enrolling adult (> 18 years) patients previously diagnosed with SUDD between 2010 and 2022. The diagnosis was confirmed by retrospective clinical evaluation and based on persistent abdominal pain, particularly in the lower left abdomen, and any imaging evidence of colonic diverticula, in accordance with recent international guidelines [10]. Patients without these diagnostic features were excluded. In order to retrieve the largest amount of data and prevent potential diagnostic and data collection biases, we exclusively included in the analysis those SUDD patients who had undergone at least one recent gastroenterological outpatient visit (since 2018, the first year with digital report availability in our centre) and had participated in telephonic interviews conducted in May 2023.

All patient data were extracted from medical records and missing data were retrieved through the telephonic follow-up.

The primary endpoint of the study was to estimate the overall, patient-dependant and physician-dependant diagnostic delay. As a secondary aim, we assessed potential factors associated with greater diagnostic delay.

### Socio-demographic and clinical data

For each patient, comprehensive socio-demographic data and risk factors for diverticular disease were collected, including age, sex, familiar history of diverticular disease, smoking habit (i.e., including active smokers, irrespective of the number of cigarettes/day, and smoke quitters for less than 5 years), fibres intake (< 10 estimated total grams of fibre per day was considered as low dietary fibres intake), alcohol consumption (more than 2 drink/day for men and 1 drink/day for women), bowel movement according to Rome IV criteria, comorbidities (including any clinically significant comorbidity -i.e. neoplastic, gastrointestinal, cardiovascular, neurological and rheumatological disorders that require specific treatment and impact patient outcomes-, and all cardiovascular and gastrointestinal comorbidities), previous use of non-steroidal anti-inflammatory drugs, steroids and opiates, history of abdominal surgery, exercise habit (at least 2 h of moderate physical activity/week), body mass index (BMI), years of education, socioeconomic status and exemption from healthcare taxes. Disease-related details at diagnosis were evaluated, i.e. disease localization and the need for hospitalization. Furthermore, data regarding patient outcomes were assessed, namely progression to diverticulitis, hospitalization, need for surgery and death for diverticular disease.

The assessment of diagnostic delay involved several data points, including the time of diagnosis, the time elapsed between the first onset of symptoms or signs clearly related to SUDD and the final diagnosis (defined overall diagnostic delay), the time elapsed between the onset of disease and the first referral to a physician, either general practitioner or specialist, (defined patient-dependant diagnostic delay), the time elapsed between the first medical consultation and the final diagnosis (defined physician-dependant diagnostic delay), previous misdiagnoses and the number of physicians consulted before reaching the final diagnosis of SUDD.

### Statistical analysis

The statistical analysis was conducted using Stata 17 (StataCorp, College Station, TX, USA). We use median and interquartile range (IQR) to describe continuous data, while categorical data were presented as counts and percentages. Missing data were excluded from statistical calculations. Chi-squared test and Mann–Whitney test were employed to assess the association between the 75th percentile of diagnostic delay (overall, patient-dependant, and physician-dependant) and relevant variables. A two-sided p-value less than 0.05 was considered statistically significant. Multivariate analysis was performed using logistic regression models, including non-collinear variables with a p-value less than 0.2 in the univariable analysis. Highly correlated predictors with a p-value less than 0.01 in the univariable analysis were removed from multivariable models to minimize overfitting. The study was approved by the local Ethics Committee (2016, Protocol number 004820) and patients provided written informed consent prior to study participation.

## Results

### Patients enrolled and diagnostic delay

**Fig. 1:**
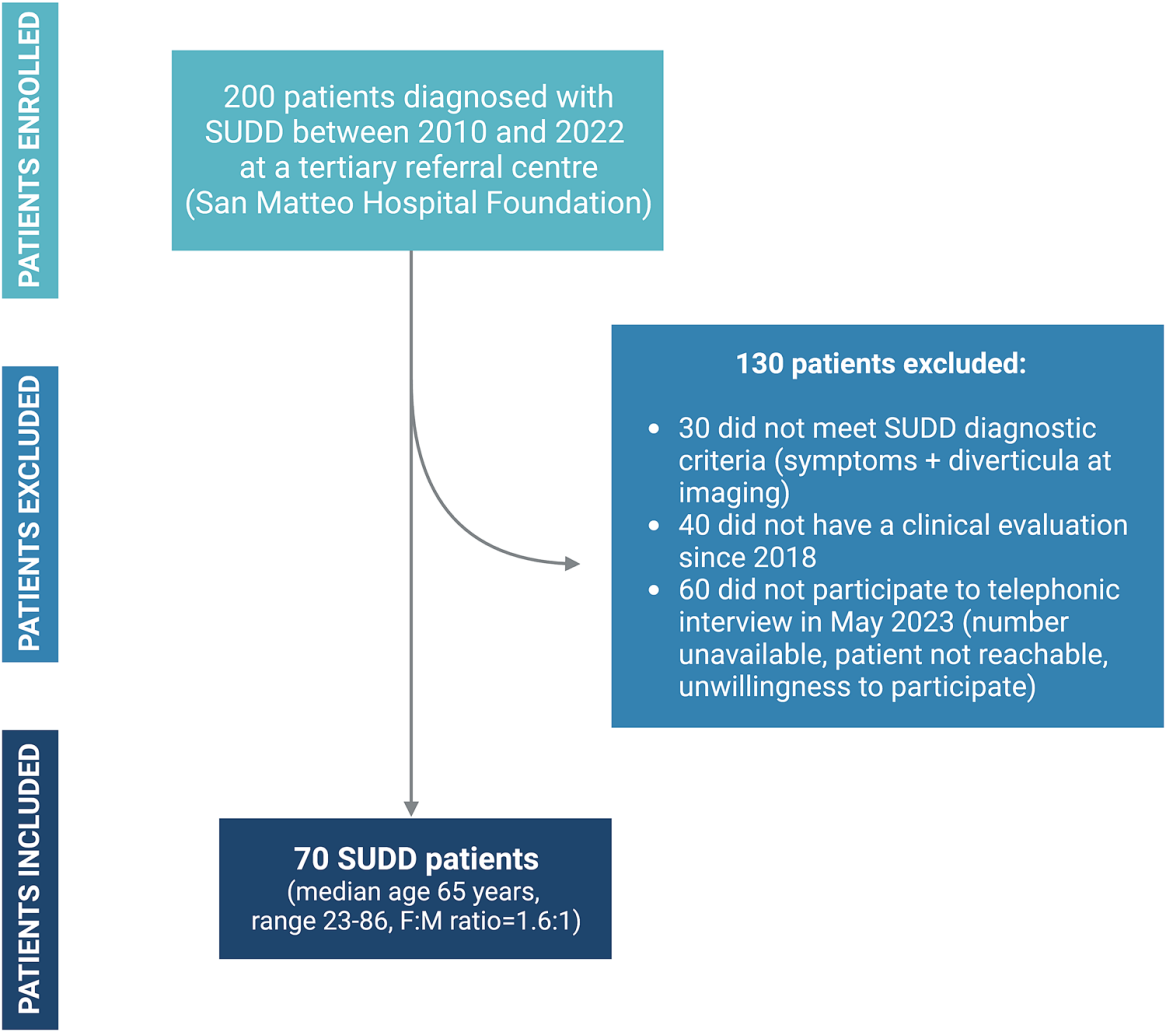
Flow-chart of patient enrolment and inclusion process. The figure illustrates the process of retrospective patient enrolment and inclusion in the study. Patients were excluded if they did not meet SUDD diagnostic criteria (symptoms + evidence of diverticula on imaging), had not been clinically evaluated since 2018, and did not participate in the May 2023 follow-up calls. Created with “Biorender.com”. *SUDD, symptomatic uncomplicated diverticular disease*

A total of 200 patients with a SUDD diagnosis were retrospectively enrolled in the study. According to inclusion criteria, 70 patients (median age 65 years, range 23–86, F:M ratio = 1.6:1) were considered in the analysis. The flow-chart showing the enrolment and inclusion of patients in the study analysis is presented in Fig. 1. In Table 1 socio-demographic and clinical data of study population at diagnosis are reported. In this study population a high prevalence of any clinically significant, cardiovascular and gastrointestinal comorbidities was found (64%, 41% and 76%, respectively). Of note, the 47% of patients had a sigmoid colon localization of diverticula, while 45% had multiple localization. Only 2 patients needed hospitalization at diagnosis.

**Table 1:** Socio-demographic and clinical features of study population at the time of diagnosis

	SUDD patients
Patients n. (%)	70 (100)
Age (median) [IQR]	65 [52–74]
F/M (ratio)	43/27 (1.6:1)
Familiar history of diverticular disease (%)	13 (20)
Smoking habit (%)	14 (21)
Low dietary fibres intake (%)	6 (10)
Alcohol consumption (%)	26 (41)
Bowel movement-Rome IV-(%)	
Constipation	16 (23)
Diarrhoea	20 (29)
Mixed	16 (23)
Comorbidities (%)	
Any clinically significant	45 (64)
CV	29 (41)
GI	53 (76)
Previous NSAIDs/steroid use (%)	13 (20)
Previous opiates use (%)	3 (5)
Previous abdominal surgery (%)	28 (41)
Exercise (%)	32 (52)
BMI (median) [IQR]	24 [22–27]
Education > 13 years (%)	24 (37)
Income > 1000 €/month (%)	46 (73)
Exemption from healthcare taxes (%)	25 (42)
Diverticula localisation (%)	
Sigmoid colon	29 (47)
Other localization	5 (8)
Multiple localization	28 (45)
Hospitalisation (%)	2 (3)

Missing data were excluded from statistical calculations

BMI, body mass index; CV, cardiovascular; F, female; NSAIDs, non-steroidal anti-inflammatory drug; GI, gastrointestinal; IQR, interquartile range; M, male; SUDD, symptomatic uncomplicated diverticular disease

**Fig. 2:**
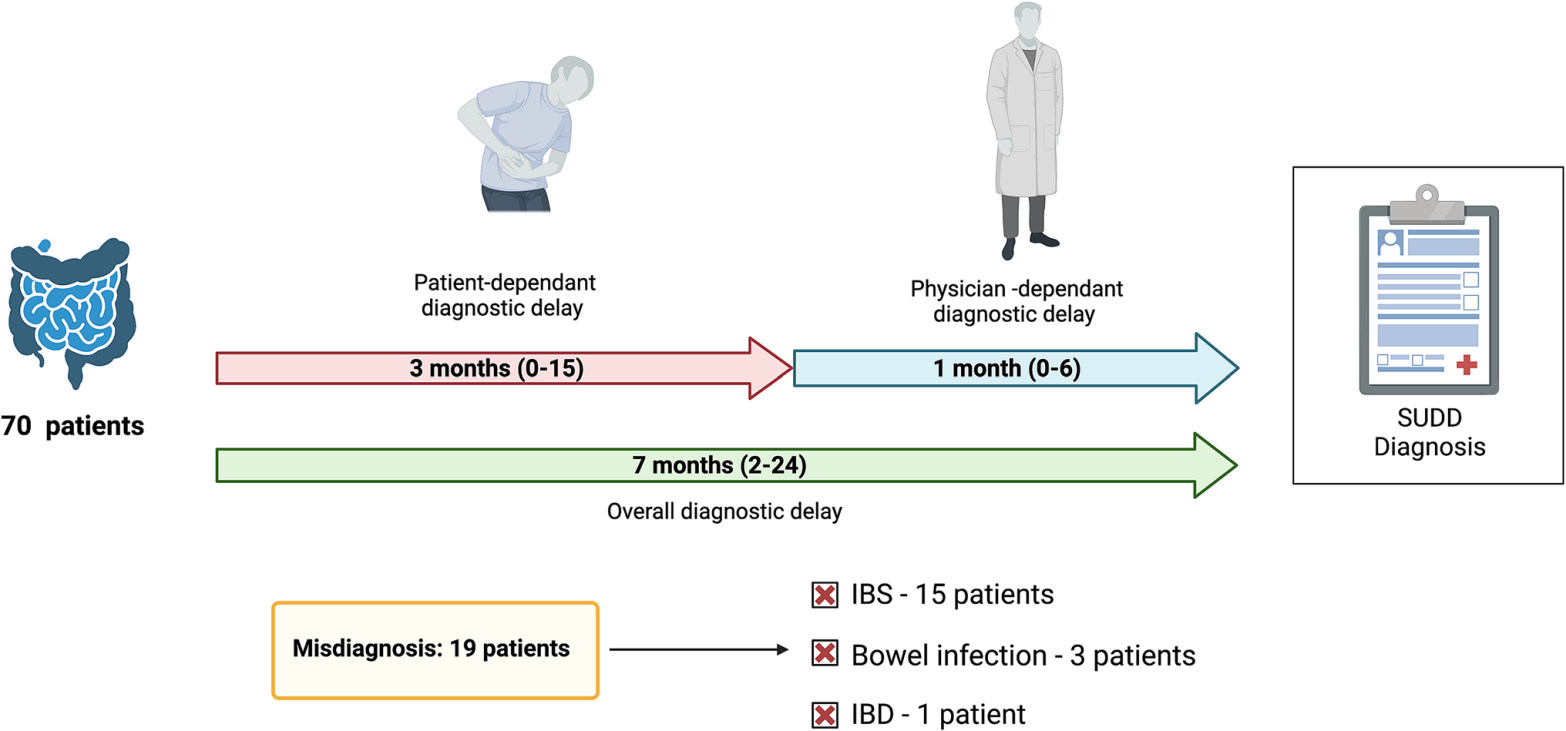
Diagnostic delay in symptomatic uncomplicated diverticular disease. The overall, patient-dependant and physician-dependant diagnostic delay in 70 patients with symptomatic uncomplicated diverticular disease is schematically represented. Values are expressed as median and interquartile range. Misdiagnosis are reported. Created with “Biorender.com”. *IBD, inflammatory bowel disease; IBS, irritable bowel syndrome; SUDD, symptomatic uncomplicated diverticular disease*

As represented in Fig. 2 and reported in Table 2, the median overall diagnostic delay was 7 months (IQR 2–24), the patient-dependant delay was 3 months (IQR 0–15) and the physician-dependant delay was 1 month (IQR 0–6). 19 patients had a previous misdiagnosis, namely IBS in 15 patients, bowel infection in 3 patients and IBD in 1 patient. Moreover, the 35% of patients consulted 2 or more physician before the final diagnosis of SUDD.

**Table 2:** Diagnostic delay, previous misdiagnosis and physicians consulted before the definitive diagnosis

	SUDD patients
Patients n. (%)	70 (100)
Diagnostic delay	
Overall DD months (median) [IQR]	7 [2–24]
Patient-dependant DD months (median) [IQR]	3 [0–15]
Physician-dependant DD months (median) [IQR]	1 [0–6]
Previous misdiagnosis (%)	
IBS	15 (25)
Bowel infection	3 (5)
IBD	1 (2)
None	42 (69)
Physician consulted until diagnosis ≥ 2 (%)	22 (35)

Missing data were excluded from statistical calculations

DD, diagnostic delay; IBD, inflammatory bowel; IBS, irritable bowel syndrome; IQR, interquartile range; SUDD, symptomatic uncomplicated diverticular disease

### Univariate analysis for factors affecting diagnostic delay

#### Overall diagnostic delay

A high overall diagnostic delay was defined as a delay greater than or equal to 24 months, corresponding to the 75th percentile of delay. In the univariate analysis (Supplementary Table 1), the previous misdiagnosis was significantly associated with an overall diagnostic delay ≥ 24 months (p = 0.01). In particular, IBS was found in 53% of patients with an overall diagnostic delay ≥ 24 months while in just 14% of patients with a delay < 24 months. Additionally, a significant correlation was found for the education greater than 13 years (p = 0.03) and the number of physicians consulted before diagnosis ≥ 2 (p < 0.01). Of note, younger age was significantly associated with a longer overall diagnostic delay (p = 0.03).

#### Patient-dependant diagnostic delay

A patient-dependant diagnostic delay greater than or equal to 15 months, corresponding to the 75th percentile, was considered as a long delay. At univariate analysis (Supplementary Table 2), consulting 2 or more physicians was significantly associated with a longer patient-dependant diagnostic delay (p = 0.03). A trend towards statistical significance was found for higher education (p = 0.07).

#### Physician-dependant diagnostic delay

A physician-dependant diagnostic delay greater than or equal to 6 months, corresponding to the 75th percentile, was considered a long delay. The univariate analysis (Supplementary Table 3) showed a significant correlation between a longer physician-dependant diagnostic delay and previous misdiagnosis (p = 0.01), as well as the number of physician consulted ≥ 2 (p < 0.01). A trend towards statistical significance was found for smoking habit (p = 0.05) and previous GI comorbidities (p = 0.07).

### Multivariable analysis for factors affecting diagnostic delay

Table 3 displays the results of multivariable analyses fitted to the 75th percentile of diagnostic delay, for factors affecting the overall, patient-dependant and physician-dependant diagnostic delay. A previous misdiagnosis proved to be a risk factor for a greater overall (OR 9.99, p = 0.01) and physician-dependant (OR 6.46, p = 0.02) diagnostic delay. Moreover, higher education was identified as a risk factor for a longer overall diagnostic delay (OR 8.74, p = 0.02). On the other hand, a history of previous abdominal surgery was found to be a protective factor for the physician-dependant diagnostic delay (OR 0.19, p = 0.04). Lastly, consulting two or more physicians before SUDD diagnosis was confirmed as a risk factor for patient-dependant diagnostic delay (OR 4.73, p = 0.03). No significant associations were observed for the other variables considered.

**Table 3:** Multivariate analysis for factors affecting diagnostic delay

	Odds ratio	95% C.I	*p*
*Overall DD*			
Age	1.01	− 0.062–0.072	0.87
CV comorbidities	0.34	− 3.149–0.675	0.26
BMI	0.84	− 0.434–0.054	0.15
Education > 13 years	8.74	0.476–4.175	**0.02**
Previous misdiagnosis	9.99	0.574–4.397	**0.01**
*Patient-dependant DD*			
Age	0.99	− 0.971–0.435	0.66
Alcohol consumption	1.59	− 1.002–1.949	0.53
Education > 13 years	1.51	− 1.211–2.011	0.61
Physician consulted until diagnosis ≥ 2 (%)	4.73	0.179–3.034	**0.03**
*Physician-dependant DD*			
Smoking habit	2.87	− 3.977–6.164	0.38
GI comorbidities	3.74	− 0.673–4.364	0.26
Previous abdominal surgery	0.19	− 3.414–0.119	**0.04**
BMI	0.87	− 0.351–0.041	0.17
Previous misdiagnosis	6.46	0.352–3.636	**0.02**

Missing data were excluded from statistical calculations. Variables that reach statistical significance (p<0.05) are presented in bold

MI, body mass index; CI, confidence interval; CV, cardiovascular; DD, diagnostic delay; GI, gastrointestinal

### Patients’ outcomes and diagnostic delay

During the follow-up (median 5 years, IQR 4–8), a total of 8 patients (11%) experienced progression to diverticulitis and required hospitalisation due to diverticular disease, only 2 patients underwent surgery, and no deaths were reported (see Table 4). When stratified according to the overall diagnostic delay, a higher frequency of progression to diverticulitis and hospitalisation for diverticular disease (17% vs 10%) was seen in patients with overall diagnostic delay higher than 24 months, though not statistically significant (p = 0.42).

**Table 4:** Patients’ outcomes at follow-up and overall diagnostic delay ≥ 24 months

	SUDD patients	DD < 24 months	DD ≥ 24 months	p
Patients n. (%)	70 (100)	52 (75)	18 (25)	–
Follow-up, median (years; IQR)	5 [4–8]	5 [4–9]	5 [3–8]	–
Progression to diverticulitis (%)	8 (11)	5 (10)	3 (17)	0.42
Hospitalisation (%)	8 (11)	5 (10)	3 (17)	0.42
Surgery (%)	2 (3)	1 (2)	1 (6)	0.43
Death (%)	0	0	0	–

*DD, diagnostic delay; IQR, interquartile range*

## Discussion

This single-centre retrospective study aimed to assess the diagnostic delay in patients with SUDD. To the best of our knowledge, there are no available data on the literature regarding this topic. Our study revealed a substantial overall diagnostic delay, secondary to both patients and physicians. Considered the SUDD burden on patients’ quality of life and the possible risk of disease progression and complications, this delay could have a significant impact on both patients and health-care systems. Therefore, more effort should be made to minimise delayed diagnosis. Indeed, an earlier SUDD diagnosis enables prompt initiation of available treatments and dietary/lifestyle modifications, leading to a significant improvement in symptom management and quality of life. Additionally, this could also potentially prevent complications on a long term.

Notably, the misdiagnosis emerged as one of the main risk factors for both overall and physician-dependant diagnostic delay. This expected result could be explained by the general tendency to consider correct the diagnosis already formulated despite contrary evidence, the so-called “anchoring bias” [11]. In particular, 25% of our patients received a misdiagnosis of IBS. Furthermore, our study showed a significant correlation between consulting two or more physicians and increased diagnostic delay. Taken together, these results suggest that improving the challenging differential diagnosis between SUDD and IBS is a crucial step to reduce diagnostic delay [12, 13].

Although SUDD and IBS share several clinical features and their potential overlap is still a matter of debate among experts [14, 15], some key features can aid in the differential diagnosis. First of all, the type of abdominal pain can provide a valuable hint in the diagnostic process. Tursi et al. suggested that moderate to severe and prolonged left lower-abdominal pain is more commonly associated with SUDD and can help differentiate it from IBS [16]. Furthermore, the evaluation of risk factors for diverticular disease during the diagnostic process is of paramount importance. Several factors, including lifestyle, dietary habits, smoking and alcohol use, contribute to the development of diverticular disease and their presence should guide physicians’ diagnostic reasoning [17]. Our results suggest that a previous abdominal surgery is usually taken into consideration by clinicians, as it demonstrated a significant protective effect on physician-dependant diagnostic delay at multivariate analysis. Nonetheless, other factors did not display the same trend. The proportion of patients with smoking habit and GI comorbidities was higher in the group with a high physician-dependant diagnostic delay, exhibiting a trend towards significance at univariate analysis. A comprehensive clinical evaluation focusing on risk factors associated with diverticular disease is therefore imperative. However, such evaluation must be critical and cautious, as the absence of red flags for diverticular disease should not lead to an incorrect a priori exclusion of SUDD diagnosis. For instance, even if diverticular disease is more common in older patients, more caution should be exercised when evaluating younger patients. Our results showed that younger patients had a significant prolonged overall diagnostic delay at univariate analysis compared with older patients.

Thus, anamnestic evaluation is a cornerstone in diagnosing diverticular disease, but additional objective tools are needed to avoid diagnostic errors. Faecal calprotectin, for instance, has shown good efficacy in differentiating SUDD from IBS, being positive in the majority of patients with SUDD and absent in patients with IBS [16]. Cross-sectional imaging examinations, such as abdominal ultrasound, have also garnered interest [18]. This fast and cost-effective technique could serve as a useful tool in the challenging differential diagnosis between organic and functional disease [19]. The absence or presence of diverticula, along with features like *muscularis propria* thickening, could guide the diagnostic work-up [20]. Moreover, using colonoscopy as the gold standard, intestinal ultrasound exhibited a high level of accuracy in detecting colonic diverticula, achieving a sensitivity of 96% and a specificity of 98.5% [20]. The wise combination of clinical information and objective diagnostic tools could help in differentiating SUDD from IBS, leading to reduced diagnostic delay and a prompt therapeutic approach.

Even if there is no consensus on the optimal management for SUDD and a significant overlap with IBS treatment exists [21], a long-term approach with rifaximin/mesalamine and probiotics may be recommended to relieve symptoms and prevent progression to diverticulitis. A meta-analysis by Bianchi et al. showed efficacy of rifaximin plus fibre supplementation in symptom relief and prevention of complications at 1 year [22]. Another randomized clinical trial showed significant effect of cyclic mesalamine and probiotics in maintaining remission [23]. Given the influence of diet and lifestyle factors on the risk of diverticular disease, promoting a healthy lifestyle is of paramount importance. Indeed, a recent study showed that men who adhered to a low-risk lifestyle, which included maintaining a healthy BMI, consuming more than 23 g of fibre per day, limiting red meat consumption, engaging in regular physical exercise and refraining from smoking, experienced a 75% reduction in the risk of diverticulitis [24]. All in all, it is crucial to adopt a comprehensive and personalised approach to maintain remission, which should be a primary clinical goal in SUDD management to prevent disease progression and complications. In our study population, 11% of patients experienced disease progression to diverticulitis, leading to hospitalisation. Furthermore, 2 patients underwent surgery and there were no reported deaths. A higher diagnostic delay was associated with a greater frequency of negative outcomes, even if no statistical difference was found. While these finding are limited by the relatively small number of patients and outcomes in the study, they underscore the importance of a prompt diagnosis in reducing the onset of disease complications. Nonetheless, further evaluation through prospective studies with larger populations is warranted, also to evaluate the impact of diagnostic delay on patients’ quality of life.

Lastly, it is noteworthy that having an advanced scholastic education was a significant risk factor for greater diagnostic delay. This finding could potentially stem from a tendency to underestimate symptoms and a lack of knowledge about complications associated with gastrointestinal disorders, especially among patients with an advanced educational background. This finding highlights the importance of enhancing awareness among the general population about these conditions, with the aim of reducing patient-dependant diagnostic delay.

Our study has some limitations. First, it is a retrospective study and, therefore, suffers from bias related to data collection, as not all data were consistently available in clinical records. We tried to overcome this limitation by selecting patients with recent follow-up and retrieving missing data through telephone calls. Moreover, this is a single-centre study involving a selected population evaluated in an outpatient setting of a tertiary referral hospital, which may have introduced a selection bias. The COVID-19 pandemic may have also influenced our results, as it could have caused a diagnostic delay in SUDD patients, as demonstrated for other chronic disorders [25]. Not least, our SUDD population had a high prevalence of complex patients, potentially leading to underrepresentation of patients encountered in general practice and other settings. Therefore, larger prospective studies encompassing patients from different settings are warranted.

To conclude, our study revealed a notable diagnostic delay, both patient- and physician-dependant, experienced by patients with SUDD. More efforts should be made to reduce this delay, preventing disease progression and complications. To address the physician-dependant diagnostic delay, increased attention should be given to the challenging differential diagnosis with IBS. A comprehensive patient evaluation, integrating diagnostic tools such as faecal calprotectin and intestinal ultrasound, should be performed, especially in individuals with risk factors for diverticular disease. As concerns patients, it is important to enhance awareness of this condition, enabling timely recognition of symptoms and appropriate medical referral.

### Supplementary Information

Below is the link to the electronic supplementary material.

Supplementary Table 1. Univariate analysis for factors affecting an overall diagnostic delay ≥ 24 months. Supplementary Table 2. Univariate analysis for factors affecting a patient-dependant diagnostic delay ≥ 15 months. Supplementary Table 3. Univariate analysis for factors affecting a physician-dependant diagnostic delay ≥ 6 months.

## Data Availability

The data that support the findings of this study are available from the corresponding author upon reasonable request.
