# Supplementary material for: Diagnostic delay in symptomatic uncomplicated diverticular disease: an Italian tertiary referral centre study
Source: Intern Emerg Med. 2023 Oct 27;19(1):99–106. doi: 10.1007/s11739-023-03446-x (PMC10827944; doi:10.1007/s11739-023-03446-x)
Supplement: Supplementary file 1 — Supplementary Table 1. Univariate analysis for factors affecting an overall diagnostic delay ≥ 24 months. Supplementary Table 2. Univariate analysis for factors affecting a patient-dependant diagnostic delay ≥ 15 months. Supplementary Table 3. Univariate analysis for factors affecting a physician-dependant diagnostic delay ≥ 6 months. [file 11739_2023_3446_MOESM1_ESM.docx]

**Supplementary Table 1.** Univariate analysis for factors affecting an overall diagnostic delay ≥ 24 months

|  | **DD < 24 months** | **DD ≥ 24 months** | **p** |
| --- | --- | --- | --- |
| Patients n. (%) | 52 (75) | 18 (25) | - |
| Age (median) [IQR] | 67 [56-77] | 53 [44-71] | **0.03** |
| F/M (ratio) | 31/21 (1.5:1) | 12/6 (2:1) | 0.59 |
| Familiar history of diverticular disease (%) | 9 (18) | 4 (25) | 0.54 |
| Smoking habit (%) | 10 (20) | 4 (24) | 0.79 |
| Low dietary fibres intake (%) | 6 (13) | 0 | 0.15 |
| Alcohol consumption (%) | 18 (38) | 8 (50) | 0.41 |
| Bowel movement -Rome IV- (%) |  |  |  |
| Constipation | 12 (24) | 4 (22) | 0.69 |
| Diarrhoea | 13 (25) | 7 (39) |  |
| Mixed | 12 (24) | 4 (22) |  |
| Comorbidities (%) |  |  |  |
| Any clinically significant | 34 (65) | 11 (61) | 0.74 |
| CV | 24 (46) | 5 (28) | 0.17 |
| GI | 38 (73) | 15 (83) | 0.38 |
| Previous NSAIDs/steroid use (%) | 10 (20) | 3 (18) | 0.80 |
| Previous opiates use (%) | 2 (4) | 1 (6) | 0.76 |
| Previous abdominal surgery (%) | 22 (43) | 6 (35) | 0.57 |
| Exercise (%) | 24 (51) | 8 (53) | 0.88 |
| BMI (median) [IQR] | 25 [23-27] | 22 [21-29] | 0.10 |
| Education > 13 years (%) | 14 (30) | 10 (59) | **0.03** |
| Income > 1000 €/month (%) | 32 (70) | 14 (82) | 0.31 |
| Exemption from healthcare taxes (%) | 20 (45) | 5 (31) | 0.32 |
| Diverticula localisation at diagnosis (%) |  |  |  |
| Sigmoid colon | 21 (47) | 8 (47) | 0.79 |
| Other localization | 3 (6) | 2 (12) |  |
| Multiple localization | 21 (47) | 7 (41) |  |
| Hospitalisation at diagnosis (%) | 2 (4) | 0 | 0.39 |
| Previous misdiagnosis (%) |  |  |  |
| IBS | 6 (14) | 9 (53) | **0.01** |
| Bowel infection | 3 (7) | 0 |  |
| IBD | 1 (2) | 0 |  |
| None | 34 (77) | 8 (47) |  |
| Physician consulted until diagnosis ≥ 2 (%) | 10 (21) | 12 (80) | **< 0.01** |

*Abbreviations: BMI ,body mass index; CV, cardiovascular; DD, diagnostic delay; F, female; NSAIDs, non-steroidal anti-inflammatory drug; GI, gastrointestinal; IBD, inflammatory bowel; IBS, irritable bowel syndrome; IQR, interquartile range; M, male.* Missing data were excluded from statistical calculations

**Supplementary Table 2.** Univariate analysis for factors affecting a patient-dependant diagnostic delay ≥ 15 months

|  | **DD < 15 months** | **DD ≥ 15 months** | **p** |
| --- | --- | --- | --- |
| Patients n. (%) | 52 (75) | 18 (25) | - |
| Age (median) [IQR] | 67 [56-77] | 55 [44-70] | 0.09 |
| F/M (ratio) | 32/20 (1.6:1) | 11/7 (1.6:1) | 0.98 |
| Familiar history of diverticular disease (%) | 9 (18) | 4 (27) | 0.44 |
| Smoking habit (%) | 10 (20) | 4 (24) | 0.79 |
| Low dietary fibres intake (%) | 5 (10) | 1 (7) | 0.61 |
| Alcohol consumption (%) | 17 (36) | 9 (56) | 0.16 |
| Bowel movement -Rome IV- (%) |  |  |  |
| Constipation | 13 (25) | 3 (18) | 0.59 |
| Diarrhoea | 13 (25) | 7 (41) |  |
| Mixed | 12 (23) | 4 (24) |  |
| Comorbidities (%) |  |  |  |
| Any clinically significant | 34 (65) | 11 (61) | 0.74 |
| CV | 23 (44) | 6 (33) | 0.42 |
| GI | 38 (73) | 15 (83) | 0.38 |
| Previous NSAIDs/steroid use (%) | 10 (20) | 3 (19) | 0.91 |
| Previous opiates use (%) | 2 (4) | 1 (6) | 0.71 |
| Previous abdominal surgery (%) | 21 (40) | 7 (44) | 0.81 |
| Exercise (%) | 24 (51) | 8 (53) | 0.88 |
| BMI (median) [IQR] | 25 [23-27] | 23 [21-29] | 0.55 |
| Education > 13 years (%) | 15 (31) | 9 (56) | 0.07 |
| Income > 1000 €/month (%) | 33 (70) | 13 (81) | 0.39 |
| Exemption from healthcare taxes (%) | 19 (42) | 6 (40) | 0.88 |
| Diverticula localisation at diagnosis (%) |  |  |  |
| Sigmoid colon | 20 (44) | 9 (53) | 0.81 |
| Other localization | 4 (8) | 1 (6) |  |
| Multiple localization | 21 (47) | 7 (41) |  |
| Hospitalisation at diagnosis (%) | 2 (4) | 0 | 0.41 |
| Previous misdiagnosis (%) |  |  |  |
| IBS | 8 (18) | 7 (41) | 0.21 |
| Bowel infection | 3 (7) | 0 |  |
| IBD | 1 (2) | 0 |  |
| None | 32 (73) | 10 (59) |  |
| Physician consulted until diagnosis ≥ 2 (%) | 13 (27) | 9 (64) | **0.03** |

*Abbreviations:* BMI ,body mass index; CV, cardiovascular; DD, diagnostic delay; F, female; NSAIDs, non-steroidal anti-inflammatory drug; GI, gastrointestinal; IBD, inflammatory bowel; IBS, irritable bowel syndrome; IQR, interquartile range; M, male. Missing data were excluded from statistical calculations.

**Supplementary Table 3.** Univariate analysis for factors affecting a physician-dependant diagnostic delay ≥ 6 months

|  | **DD < 6 months** | **DD ≥ 6 months** | **p** |
| --- | --- | --- | --- |
| Patients n. (%) | 50 (71) | 20 (29) | - |
| Age (median) [IQR] | 66 [56-76] | 60 [48-72] | 0.26 |
| F/M (ratio) | 31/19 (1.6:1) | 12/8 (1.5:1) | 0.88 |
| Familiar history of diverticular disease (%) | 8 (17) | 5 (26) | 0.39 |
| Smoking habit (%) | 7 (15) | 7 (37) | 0.05 |
| Low dietary fibres intake (%) | 6 (14) | 0 | 0.09 |
| Alcohol consumption (%) | 18 (39) | 8 (47) | 0.57 |
| Bowel movement -Rome IV- (%) |  |  |  |
| Constipation | 13 (27) | 3 (15) | 0.58 |
| Diarrhoea | 13 (27) | 7 (35) |  |
| Mixed | 10 (20) | 6 (30) |  |
| Comorbidities (%) |  |  |  |
| Any clinically significant | 33 (66) | 12 (60) | 0.64 |
| CV | 23 (46) | 6 (30) | 0.22 |
| GI | 35 (70) | 18 (90) | 0.07 |
| Previous NSAIDs/steroid use (%) | 8 (17) | 5 (26) | 0.39 |
| Previous opiates use (%) | 2 (4) | 1 (5) | 0.86 |
| Previous abdominal surgery (%) | 23 (47) | 5 (26) | 0.12 |
| Exercise (%) | 24 (52) | 8 (50) | 0.88 |
| BMI (median) [IQR] | 25 [23-27] | 23 [21-25] | 0.06 |
| Education > 13 years (%) | 17 (38) | 7 (37) | 0.94 |
| Income > 1000 €/month (%) | 32 (73) | 14 (74) | 0.94 |
| Exemption from healthcare taxes (%) | 19 (45) | 6 (33) | 0.39 |
| Diverticula localisation at diagnosis (%) |  |  |  |
| Sigmoid colon | 20 (47) | 9 (47) | 0.29 |
| Other localization | 2 (5) | 3 (16) |  |
| Multiple localization | 21 (49) | 7 (37) |  |
| Hospitalisation at diagnosis (%) | 2 (4) | 0 | 0.35 |
| Previous misdiagnosis (%) |  |  |  |
| IBS | 6 (14) | 9 (53) | **0.01** |
| Bowel infection | 3 (7) | 0 |  |
| IBD | 1 (2) | 0 |  |
| None | 34 (77) | 8 (47) |  |
| Physician consulted until diagnosis ≥ 2 (%) | 8 (18) | 14 (74) | **< 0.01** |

*Abbreviations: BMI, body mass index; CV, cardiovascular; DD, diagnostic delay; F, female; NSAIDs, non-steroidal anti-inflammatory drug; GI, gastrointestinal; IBD, inflammatory bowel; IBS, irritable bowel syndrome; IQR, interquartile range; M, male.* Missing data were excluded from statistical calculations.
